# The collagen‐augmented chondrogenesis technique demonstrates superior cartilage repair compared to microfracture for cartilage defects of the knee joint, regardless of age

**DOI:** 10.1002/ksa.12500

**Published:** 2024-10-06

**Authors:** Man Soo Kim, Keun Young Choi, Ryu Kyoung Cho, Hyuk Jin Jang, Dong Ho Kwak, Sung Cheol Yang, Seung Taek Oh, Yong In

**Affiliations:** ^1^ Department of Orthopaedic Surgery, Seoul St. Mary's Hospital, College of Medicine The Catholic University of Korea Seoul Korea

**Keywords:** age, cartilage, chondrogenesis, collagen, knee, microfracture

## Abstract

**Purpose:**

This study investigated whether age affects clinical outcomes and cartilage repair quality in patients who underwent collagen‐augmented chondrogenesis.

**Methods:**

The study included patients who underwent either the collagen‐augmented chondrogenesis technique or microfracture for cartilage defects of the knee joint of International Cartilage Repair Society grade 3 or 4. Patients were categorised according to an age threshold of 50 years and the treatment method, whether collagen‐augmented chondrogenesis technique or microfracture. Group 1 comprised 31 patients aged 50 years or older who received the collagen‐augmented chondrogenesis technique, Group 2 consisted of 32 patients under the age of 50 years who received the collagen‐augmented chondrogenesis technique and Group 3 included 243 patients aged 50 years or older who received microfracture. Clinical outcomes were assessed using the walking visual analogue scale (VAS) for pain and the Western Ontario McMaster University Osteoarthritis Index scale score (WOMAC) two years after surgery. For patients with magnetic resonance imaging results 1 year postoperatively (Group 1: 30 patients; Group 2: 31 patients; and Group 3: 31 patients), Magnetic Resonance Observation of Cartilage Repair Tissue (MOCART) assessment was used to evaluate repaired cartilage lesions.

**Results:**

There were no significant differences in the VAS and WOMAC scores between the three groups 2 years after surgery (all n.s.). The MOCART score in patients who underwent MRI at 1 year postoperatively showed significant differences in the degree of defect repair, integration with the border zone, surface of the repaired tissue, adhesion and total score among the three groups (all *p* < 0.05). Post hoc analysis revealed no difference in the total MOCART scores between Groups 1 and 2. However, Groups 1 and 2 had significantly higher MOCART scores than Group 3 1 year after surgery (all *p* < 0.05).

**Conclusion:**

The collagen‐augmented chondrogenesis technique group showed improved quality of cartilage repair compared to the microfracture group, regardless of patient age. Compared with simple microfracture treatment, there were no differences in clinical outcomes between the patient groups, related to age.

**Level of Evidence:**

Level Ⅲ.

**Abbreviations**
HTO: high tibial osteotomy
ICRS: International Cartilage Repair Society
MCID: the minimal clinically important difference
MOCART: MRI observation of the cartilage repair tissue
MRI: magnetic resonance imaging
OA: osteoarthritis
VAS: visual analogue scale
WOMAC: Western Ontario McMaster University Osteoarthritis Index scale

## INTRODUCTION

Articular cartilage lesions are found in approximately 60% of the patients undergoing knee arthroscopy, although the severity of articular cartilage lesions varies [45]. Treating these cartilaginous lesions is challenging for orthopaedic surgeons [36]. Joint cartilage has poor healing potential because it is a hypocellular tissue with poor vasculature and lymphatic drainage [36]. Various methods for treating articular cartilage lesions, such as microfracture treatment, abrasion arthroplasty, osteochondral autografting and autologous chondrocyte implantation (ACI), are available [20]. Among these treatment methods, microfracture treatment is the most common and popular [27].

Microfracture treatment has been recognised and adopted as a routine procedure for articular cartilage lesions over the past 20 years [17, 21]. Despite its long history and popularity, the quality of repaired articular cartilage after microfracture remains variable and uncertain [17, 21]. The repaired cartilage is commonly known as fibrocartilage rather than hyaline cartilage [17, 21]. Furthermore, the functional outcome of microfracture treatment gradually decreases through tissue degradation over time [4, 13].

Methods for treating the knee cartilage have been developed to overcome these limitations [18, 20]. Advanced chondrogenesis techniques, such as the collagen‐augmented chondrogenesis technique, are examples of such methods [18, 20]. However, many factors determine the prognosis of a knee cartilage repair procedure [34, 44]. Age is the most important factor determining the success of treatment [34, 44], and ageing is known to negatively affect articular cartilage by impairing the healing potential [7]. Several studies have investigated the relationship between the outcomes of microfracture treatment and age, and cutoff ages have been suggested for more ambitious regenerative procedures for cartilage [9, 13]. The cutoff age is often set at 50 years [9, 13].

Little research has been conducted on the effect of age on the outcomes of advanced chondrogenic techniques, including collagen‐augmented chondrogenesis. Thus, this study investigated whether age affects clinical outcomes and cartilage repair quality in patients who underwent the collagen‐augmented chondrogenesis technique. We hypothesised that age would not affect the clinical outcome or cartilage repair quality of the collagen‐augmented chondrogenesis technique. Additionally, we hypothesised that regardless of age, superior clinical outcomes and cartilage repair quality would be achieved with the collagen‐augmented chondrogenesis technique compared to microfracture.

## METHODS

The Hospital Ethics Committee and Internal Review Board of Seoul St. Mary's Hospital approved this retrospective cohort study (KC22RASI0584). From 2014 to 2020, patients who underwent collagen‐augmented chondrogenesis technique or microfracture treatment for International Cartilage Repair Society (ICRS) grade 3 or 4 cartilage defects of the medial femoral condyle of the knee joint were investigated at a single centre. The inclusion criterion for the collagen‐augmented chondrogenesis technique or microfracture treatment was <65 years without ligament instability. Patients who underwent concomitant corrective knee osteotomies for varus deformities were also included. The exclusion criteria were as follows: inflammatory arthritis, osteonecrosis, flexion contracture greater than 15°, range of motion less than 120°, history of knee joint infection and a follow‐up period of less than 2 years. Seventy‐two patients underwent collagen‐augmented chondrogenesis, and 258 patients underwent microfracture treatment. Nine patients who underwent the collagen‐augmented chondrogenesis technique were excluded, including seven patients lost to follow‐up and two patients who underwent other knee joint surgeries during the follow‐up period. Twelve microfracture patients were excluded, including eight lost to follow‐up and four patients who underwent other surgeries on the knee joint during the follow‐up period. In this study, 50 years was considered the age threshold. Only patients 50 years older were included in the study in the microfracture group. Thus, 12 patients under the age of 50 years who underwent microfracture treatment were excluded. Finally, 63 patients with collagen‐augmented chondrogenesis and 234 patients with microfractures met the inclusion criteria.

**Figure 1:**
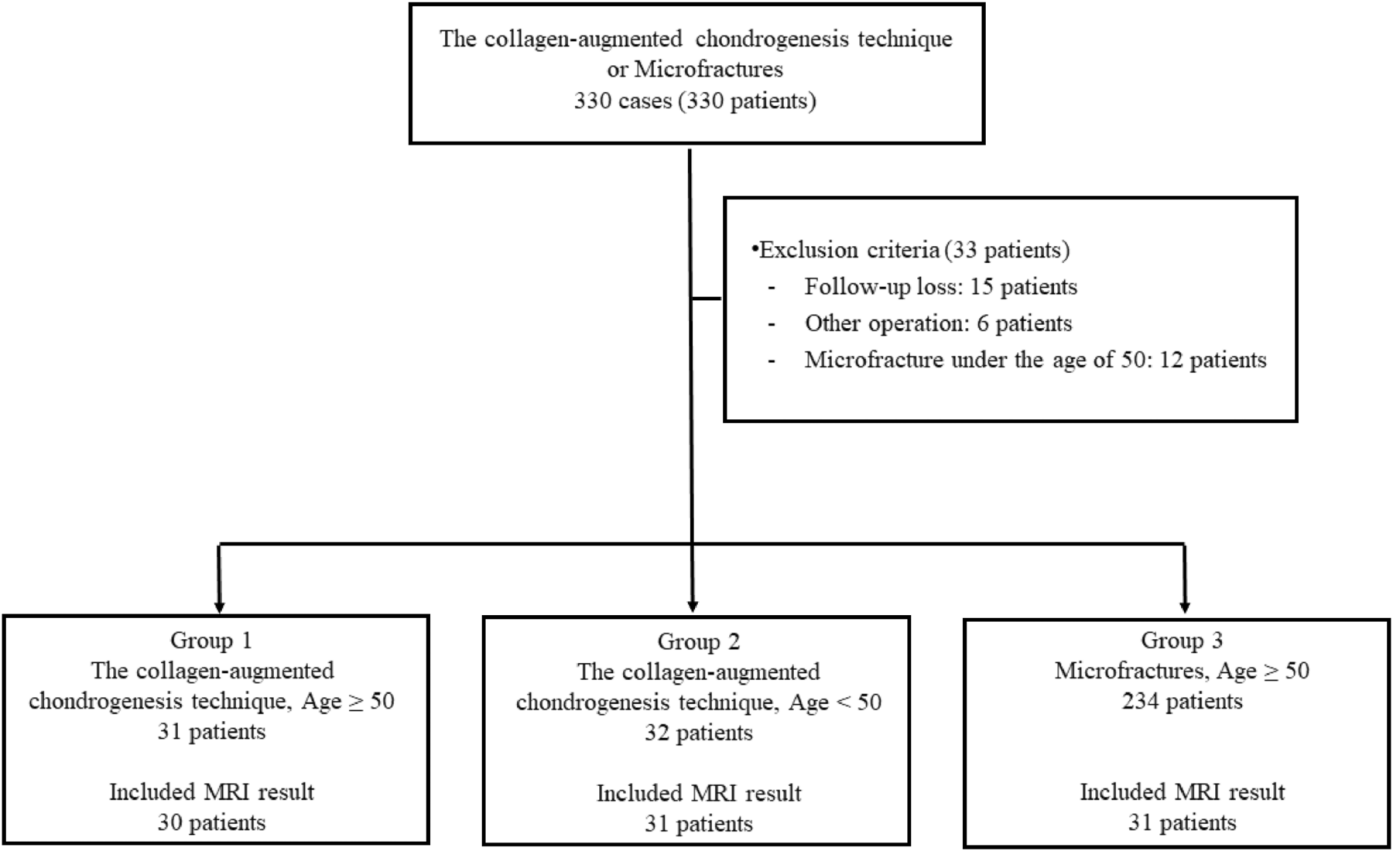
Flow chart of group allocation.

In this study, a threshold age was set to investigate the effect of age on the clinical outcomes of collagen‐augmented chondrogenesis. The age threshold was set at 50 years based on a previous study that found that the clinical results of microfracture treatment were poor after 50 years of age [9]. Group 1 comprised 31 patients aged 50 years or older who received the collagen‐augmented chondrogenesis technique, Group 2 comprised 32 patients under 50 years who received the collagen‐augmented chondrogenesis technique and Group 3 comprised 234 patients aged 50 years or older who received microfracture treatment (Figure 1).

For microfracture treatment, arthroscopic debridement was performed to remove unstable cartilage flaps. The calcified layer at the base of the lesion was removed using a curette. Microfracture holes were created at 3–4 mm. Deep subchondral drilling of more than 6 mm was performed to increase the treatment effect of microfractures, using a K‐wire instead of the general microfracture technique using an awl [24]. In the collagen‐augmented chondrogenesis technique, the implementation of microfracture treatment was the same. Arthroscopic examination and microfracture treatment to stimulate the bone marrow were performed as standard procedures in all patients. In the collagen‐augmented chondrogenesis technique, a syringe was filled with a thrombin solution, which was then transferred to a collagen implant syringe for mixing. A 1‐mL syringe was filled with 0.9 mL atelocollagen (CartiFill; SewonCellontech, Seoul, Korea) and 0.1 mL of thrombin (50 IU) mixture. Another syringe was filled with 1 mL of fibrinogen (Greenplast; Green Cross Co., Yongin, Korea). The two syringes were assembled using a mixing catheter connected to a 20‐gauge needle. The microfracture site was prepared in a dry environment using gauze. The mixture was then slowly injected into the microfracture site. Approximately 5 min after the injection, the stability of the atelocollagen layer was verified through flexion and extension of the knee [18]. When correction was necessary due to a varus deformity, a high tibial osteotomy (HTO) was performed, and the target point was set as the Fujisawa point [18].

The same postoperative rehabilitation protocol was applied to all patients who underwent collagen‐augmented chondrogenesis techniques and microfractures. Joint exercises were initiated using a continuous passive motion machine for 30 min, four times daily, starting the day after surgery. Range‐of‐motion exercises began at a joint angle of 60° and were increased by 5–10° daily. Only partial weight‐bearing ambulation was allowed for the first four weeks after surgery. Full weight‐bearing ambulation was permitted at 6 weeks postoperatively.

An independent investigator assessed clinical outcomes annually using the 100‐mm visual analogue scale (VAS) for pain and the Western Ontario and McMaster Universities Arthritis Index (WOMAC). All included patients were followed up for at least 2 years postoperatively. The WOMAC is a widely used, validated and disease‐specific questionnaire for evaluating outcomes after knee cartilage surgery. It has three subscales: pain (five items), stiffness (two items) and function (17 items). The score for each item ranges from 0 to 4, with maximum combined scores of 20 for the pain subscale, eight for the stiffness subscale and 68 for the function subscale, with a total score ranging from 0 to 96. The method most commonly used to determine the outcome of orthopaedic surgery is assessing the minimal clinically important difference (MCID) [19]. The MCID represents the smallest change in outcome that the patient can identify as beneficial [19]. The MCID for knee cartilage regeneration was set at WOMAC 14.4 points according to the results of previous studies, and the MCID achievement rate was measured among the three groups [19].

Knee magnetic resonance imaging (MRI) was performed at the 1‐year follow‐up to determine efficacy objectively, and a radiological investigation was conducted to assess cartilage regeneration. The MRI observation of the cartilage repair tissue (MOCART) score was measured twice at 2‐week intervals by an independent radiologist blinded to the details of this study. The average of the measurements was used in the analysis. Reliability was assessed by intraobserver agreement using the intraclass correlation coefficient (ICC), with both intraobserver ICC values exceeding 0.8. The MOCART score is a validated evaluation tool for assessing the structural status of cartilage repair based on MRI findings. It ranges from 0 (worst) to 100 (best) points according to the evaluation of 10 items [31]. One patient in Group 1, one in Group 2 and 203 in Group 3 did not have MRI results at 1 year postoperatively. The MOCART score was evaluated in 30 patients in Group 1, 31 in Group 2 and 31 in Group 3 who had MRI results 1 year after surgery.

Safety outcomes and subjectively and objectively identified symptoms of adverse events were assessed according to the investigators' judgement regarding directly related surgical therapeutic interventions.

### Statistical analysis

As the primary outcomes, clinical outcomes, including VAS, WOMAC and MCID achievement rates, were compared among the three groups of patients. As a secondary outcome, MOCART scores using MRI were compared among the three groups of patients, excluding patients without MRI results 1 year after surgery. In studies conducted on patients with focal cartilage lesions of the knee who exhibited characteristics similar to those enrolled in this study, the WOMAC score followed a normal distribution, with a standard deviation of 17 points and an MCID of 15 points [6]. Assuming an error rate (*α*) of 5%, a power (1‐*β*) of 80% and a dropout rate of 10% during follow‐up, the required sample size for each group is calculated to be 20 participants. Given that more than 20 patients were enrolled in the three groups, the sample size per group was deemed sufficient. Descriptive analyses were based on frequencies and percentages for categorical variables and mean and standard deviations for continuous variables. The chi‐square or Fisher's exact tests for categorical variables were conducted to compare groups according to demographic data and pre‐ and postoperative values. A post hoc analysis with Bonferroni correction was used to make pairwise comparisons and confirm statistical differences between the groups. All statistical analyses were performed using the SPSS ver. 24.0 software (SPSS Inc.). Data are expressed as means ± standard deviation. Statistical significance was set at *p* < 0.05.

## RESULTS

**Figure 2:**
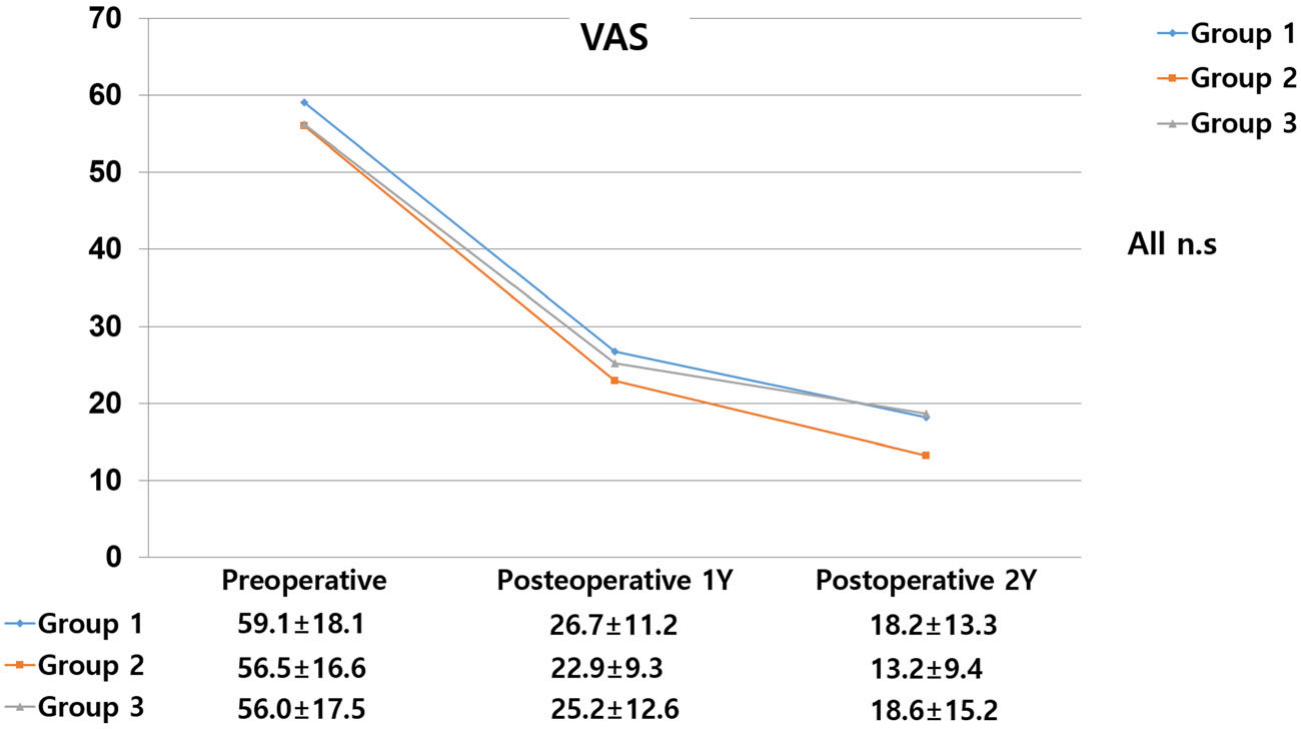
Preoperative and postoperative walking pain visual analogue scale (VAS).

The mean age was 53.0 (±4.0) years in Group 1, 40.7 (±7.8) years in Group 2 and 57.1 (±4.0) years in Group 3, with significant differences observed among the three groups (*p* < 0.05). The three groups had no significant differences in the Kellgren–Lawrence osteoarthritis (OA) grade, cartilage defect size or ICRS grade (Table 1). There were no significant differences in the VAS scores for walking pain between the three groups before surgery or at 1 and 2 years after surgery (Figure 2).

**Table 1:** Demographic and defect data of the patients.

	Group 1 (*n* = 31)	Group 2 (*n* = 32)	Group 3 (*n* = 234)	*p*‐Value
Age	53.0 ± 4.0	40.7 ± 7.8	57.1 ± 4.0	<0.001
Sex, female/male, *n*	26/5	22/10	199/35	n.s
BMI, kg/m^2^	25.7 ± 3.2	26.6 ± 3.0	25.9 ± 3.3	n.s
Operation side, right/left, *n*	12/19	14/18	115/119	n.s
Kellgren–Lawrence OA grade	2.9 ± 0.6	2.7 ± 0.5	2.8 ± 0.5	n.s
Defect size, cm^2^	4.5 ± 2.2	4.1 ± 1.9	4.4 ± 2.5	n.s
ICRS grade	3.6 ± 0.5	3.4 ± 0.5	3.6 ± 0.5	n.s
ICRS grade 3	11 (35.5%)	19 (59.4%)	96 (41.0%)	n.s
ICRS grade 4	20 (64.5%)	13 (40.6%)	138 (59.0%)	
Concomitant surgery using HTO				<0.001
HTO not performed, *n* (%)	6 (19.4%)	15 (53.1%)	12 (5.1%)	
HTO performed, *n* (%)	25 (80.6%)	17 (46.9%)	222 (94.9%)	

*Note*: Numerical variables are presented as mean ± SD. Categorical variables are presented as frequency with percentage in parentheses.

Abbreviations: BMI, body mass index; HTO, high tibial osteotomy; ICRS, International Cartilage Repair Society.

The three groups had no significant difference regarding preoperative WOMAC pain, stiffness, function subscores or total score. In addition, there was no significant difference in any of the WOMAC subscores 2 years after surgery between the three groups (all *p* < 0.05) (Table 2). As a result of dividing the clinical results of ICRS grades 3 and 4 into three groups, there was no difference in the ratio of ICRS grades 3 and 4 among the three groups. There were no differences in the clinical results between the pre‐ and postoperative WOMAC pain, function and total scores (all *p* < 0.05).

**Table 2:** The comparison of WOMAC scores.

	Group 1 (*n* = 31)	Group 2 (*n* = 32)	Group 3 (*n* = 234)	*p*‐Value
Preoperative				
Total WOMAC	44.7 ± 13.2	48.6 ± 16.6	49.9 ± 19.7	n.s
Pain	8.8 ± 3.3	9.4 ± 4.1	9.9 ± 4.3	n.s
Stiffness	3.2 ± 1.8	3.4 ± 1.8	3.9 ± 2.3	n.s
Function	32.6 ± 9.5	35.7 ± 12.2	36.1 ± 14.5	n.s
Postoperative 2 years				
Total WOMAC	23.1 ± 15.0	22.5 ± 16.5	27.3 ± 20.5	n.s
Pain	3.2 ± 2.7	4.9 ± 4.0	4.8 ± 3.9	n.s
Stiffness	2.0 ± 1.7	2.2 ± 1.8	2.5 ± 2.1	n.s
Function	17.9 ± 12.2	16.2 ± 12.0	19.6 ± 14.3	n.s

*Note*: Numerical variables are presented as mean ± SD.

Abbreviation: WOMAC, Western Ontario and McMaster Universities Arthritis Index.

The change in WOMAC score before and 2 years after surgery was 21.6 (±17.8) points in Group 1, 26.1 (±16.9) points in Group 2 and 22.6 (±25.1) points in Group 3, with no significant difference between the three groups (n. s.). The MCID achievement rate based on a WOMAC total score of 14.4 points or higher was 66.7% in Group 1, 74.2% in Group 2 and 60.7% in Group 3, with no significant difference between the three groups (n. s.).

The MOCART score at 1 year after surgery was 61.3 (±14.3) points in Group 1, 60.5 (±16.1) points in Group 2 and 47.6 (±16.1) points in Group 3, with significant differences observed between the three groups (*p* = 0.001). There were significant differences among the three groups in the degree of defect repair and filling, integration, surface area, subchondral lamina and adhesion (all *p* < 0.05) (Table 3). Post hoc analysis for identifying the relationship between the three groups showed that there was no difference between Groups 1 and 2 in terms of the degree of defect filling, surface, subchondral lamina and adhesion (all n.s.); Group 2 had results superior to those of Group 3 (all *p* < 0.05). Even in the case of integration, there was no difference between Groups 1 and 2 (all n. s.), and Group 1 showed better results than Group 3 (all *p* < 0.05). There was no difference in the total scores between Group 1 and Group 2 (n.s.), but both Groups 1 and 2 had significantly higher scores than Group 3 (all *p* < 0.05) (Table 4). No serious complications required additional hospitalisation or surgical treatment in any group.

**Table 3:** MOCART scores at 1 year postoperatively.

MOCART	Group 1 (*n* = 30)	Group 2 (*n* = 31)	Group 3 (*n* = 31)	*p*‐Value
Degree of defect repair and filling of the defect	12.8 ± 3.9	13.1 ± 5.3	9.5 ± 3.9	0.003
Integration with the border zone	11.7 ± 3.0	10.3 ± 3.1	9.0 ± 2.7	0.004
Surface of the repair tissue	7.3 ± 2.5	8.4 ± 2.4	4.0 ± 3.0	0.001
Structure of the repair tissue	2.5 ± 2.5	2.9 ± 2.5	2.4 ± 2.5	n.s
Signal intensity of the repair tissue T2‐FSE (or TSE)	11.8 ± 7.1	9.4 ± 6.7	10.0 ± 6.8	n.s
Subchondral lamina	4.3 ± 1.7	4.8 ± 0.9	3.2 ± 2.4	0.002
Subchondral bone	2.8 ± 2.5	2.9 ± 2.5	2.9 ± 2.5	n.s
Adhesions	4.2 ± 1.9	4.8 ± 0.9	3.2 ± 2.4	0.002
Effusion	4.2 ± 1.9	3.9 ± 2.1	3.2 ± 2.4	n.s
Total	61.3 ± 14.3	60.5 ± 16.1	47.6 ± 16.1	0.001

*Note*: Numerical variables are presented as mean ± SD.

Abbreviation: MOCART, MRI observation of the cartilage repair.

**Table 4:** The correlation of postoperative MOCART results between the three groups.

Dependent Variable	Generation grouping		Mean difference	*p*‐Value
Defect repair	Group 1	Group 2	−0.2	n.s
		Group 3	3.3	0.013
	Group 2	Group 3	3.5	0.006
Integration	Group 1	Group 2	1.3	n.s
		Group 3	2.6	0.002
	Group 2	Group 3	1.3	n.s
Surface	Group 1	Group 2	−1.1	n.s
		Group 3	3.3	<0.001
	Group 2	Group 3	4.4	<0.001
Structure	Group 1	Group 2	−0.4	n.s
		Group 3	0.1	n.s
	Group 2	Group 3	0.5	n.s
Signal	Group 1	Group 2	2.5	n.s
		Group 3	1.8	n.s
	Group 2	Group 3	−0.6	n.s
Subchondral lamina	Group 1	Group 2	−0.5	n.s
		Group 3	1.1	n.s
	Group 2	Group 3	1.6	0.002
Subchondral bone	Group 1	Group 2	−0.1	n.s
		Group 3	−0.1	n.s
	Group 2	Group 3	0.0	n.s
Adhesion	Group 1	Group 2	−0.5	n.s
		Group 3	1.1	n.s
	Group 2	Group 3	1.6	0.002
Effusion	Group 1	Group 2	0.3	n.s
		Group 3	0.9	n.s
	Group 2	Group 3	0.6	n.s
Total	Group 1	Group 2	0.8	n.s
		Group 3	13.7	0.003
	Group 2	Group 3	12.9	0.005

Abbreviation: MOCART, MRI observation of the cartilage repair.

## DISCUSSION

The most important finding of this study was that age did not affect the clinical outcome or the quality of repaired cartilage using the collagen‐augmented chondrogenesis technique, with the age threshold set at 50 years. Various methods have been adopted for knee cartilage regeneration; however, their effectiveness is limited by patient age [9, 25, 39]. Few reports discuss the clinical outcomes of advanced chondrogenesis techniques, including collagen‐augmented chondrogenesis technique, according to patient age. In this study, we analysed whether there was a difference in the results of the collagen‐augmented chondrogenesis technique according to age or a difference in comparison with microfracture treatment.

Several factors affect the clinical outcomes of cartilage regeneration procedures involving microfracture treatment [44]. One of these factors is age, in that it is generally known that an older patient has poorer clinical results [13, 34, 44]. However, a clear consensus regarding the cutoff age has not yet been established. The results reported for the threshold age for the effect of age on a patient's clinical outcome varied from 30 to 50 years [9, 13, 34, 44]. A survey of doctors in Turkey revealed that in microfracture treatment, the rate of surgery decreased tremendously after the age of 50 years [9]. In addition, in many studies, an age of less than 50 years was considered a criterion for inclusion in cartilage regeneration treatment [9, 13, 34, 44]. A systematic review confirmed that the clinical result of cartilage regeneration is significantly reduced in a patient aged 50 years or older [13]. Therefore, in this study, we investigated the effects of age using a threshold age of 50 years.

The observation that clinical results deteriorate with age is particularly true for microfracture treatment [25, 39], the most commonly used treatment option for cartilage regeneration [17]. In particular, the effect of age becomes more apparent with an increasing threshold of age [17, 25, 39]. Sansone et al. [39] reported the long‐term follow‐up results of abrasion arthroplasty for knee cartilage lesions. With a threshold age of 50 years, the survival rate was 89.5% for those under 50 years and 55.7% for those over 50 years, showing a significant difference. Kreutz et al. [25] examined the short‐ and long‐term results of a microfracture treatment at a threshold age of 40 years. The clinical results of microfracture treatment were poor for the group aged 40 years or older compared with those for the group younger than 40 years, and this result became even more evident with an increasing threshold age. A long‐term study of microfracture treatment of patients with knee cartilage defects under 50 years showed improved results compared to those before surgery, but the results worsened with increasing age [25, 39]. Age significantly affects healing, as a function of bone marrow‐derived mesenchymal stem cells discharged from the microfracture site deteriorates with age, reducing healing potential in older patients [10].

Some studies have shown an association between age and clinical outcomes in cartilage repair procedures involving microfracture [22, 33, 40], whereas others have found no associations [11, 12, 38]. Although different from the collagen‐augmented chondrogenesis technique, looking at autologous matrix‐induced chondrogenesis (AMIC)‐related studies using cell‐free scaffolds, the mid‐term follow‐up results for knee cartilage defects using AMIC did not show a significant correlation with age and clinical outcomes [12]. A comparison of the clinical results of AMIC for knee cartilage defects in patients aged 45 years showed no differences in IKDC, Lysholm and VAS scores between the two groups [1]. When examining the results after ACI, one study found no difference in the failure rate between the elderly and young groups [37]. In contrast, the older the patient, the higher the failure rate after microfracture or mosaic plastic surgery [40]. A recent systematic review found a negative correlation between age and clinical outcomes, including the Lysholm score, in the cartilage repair procedure [33]. This association between age and clinical outcomes in patients was also confirmed in a randomised controlled trial of 80 patients treated for ACI or microfracture, which also showed a strong association between age and clinical outcomes [22]. However, these results do not support an association between clinical outcomes and age using the collagen‐augmented chondrogenesis technique. This study investigated the impact of age on clinical outcomes and cartilage repair quality in patients undergoing collagen‐augmented chondrogenesis. This study aligns with the findings of Rosenberger et al. [37], Kon et al. [23] and Niemayer et al. [35], who reported that advanced cartilage repair techniques could yield favourable outcomes even in older patients. In this study, by including not only patients with knee cartilage defects but also patients with knee OA, the results of the collagen‐augmented chondrogenesis technique and microfracture treatments were compared according to age in patients with OA and knee cartilage defects, in contrast to previous studies [16, 32, 43]. This study obtained similar clinical results for the collagen‐augmented chondrogenesis technique, regardless of patient age. These results provide a basis for cartilage repair procedures in patients aged >50 years.

MRI is commonly used to evaluate the repair and quality of cartilage after repair [8]. It is believed that the MOCART score, which is related to the lack of defective filling, is worse for microfracture treatment than for the collagen‐augmented chondrogenesis technique because of the initial displacement of the superclot at the microfracture site without being maintained [14]. The advantages of the collagen‐augmented chondrogenesis technique include improved concentration of mesenchymal stem cells available in superclots and stability during the healing process [2, 5]. Thus, the single‐stage collagen‐augmented chondrogenesis technique is a good replacement for microfractures in the first‐line treatment of knee cartilage defects. De Windt et al. [8] reported that the MOCART total score and MOCART sub‐score for the defect‐filling item closely correlated with the clinical results. In this study, at the 2‐year follow‐up, the improved quality of the repaired cartilage observed on MRI did not correlate with the clinical results. There was no difference in the VAS, WOMAC, IKDC or KOOS scores regarding clinical results. Thus, it is necessary to investigate the correlation between improved quality and clinical results using long‐term follow‐up.

In this study, an age threshold of 50 years did not affect cartilage regeneration surgery using the collagen‐augmented chondrogenesis technique. Studies have shown favourable results with microfracture treatment, even in middle‐aged and older Asians [28]. Follow‐up observations 3 years after microfracture treatment of patients with an average age of 61 years and age range of 50–74 years showed no decline in clinical results [28]. However, other studies have shown opposite results. For instance, the follow‐up of patients with an average age of 57 years and an age range of 41–71 years for 5 years found a clear deterioration in clinical results, with approximately 34% of patients undergoing total knee arthroplasty as conversion surgery [4]. Kim et al. [17] found that in 71 patients with an average age of 51.3 years and were followed up for an average of 7.2 years, the clinical picture was best 1 year after surgery and gradually worsened until 10 years after surgery. These differences in outcomes may be due to differences in demographic data and lifestyle [16]. The follow‐up results of cartilage repair in middle‐aged patients thus vary [4, 17, 28]. In this study, there was no difference in clinical features between patients aged >50 years and those aged <50 years at the 2‐year follow‐up.

The collagen‐augmented chondrogenesis technique showed similar cartilage repair quality and clinical results in patients aged <50 years and >50 years. In addition, the collagen‐augmented chondrogenesis technique provides superior cartilage repair quality compared with microfractures, regardless of patient age. This finding suggests that age is not a limiting factor when considering collagen‐augmented chondrogenesis techniques for cartilage repair. This insight could potentially modify clinical practice by encouraging the use of the collagen‐augmented chondrogenesis technique in older patients who were previously considered suboptimal candidates for advanced cartilage repair techniques [17, 22, 33, 39]. This study adds new information to the literature by providing evidence that age did not significantly impact the clinical outcomes of the collagen‐augmented chondrogenesis technique, contrasting with the poorer outcomes often observed with microfracture in older patients [16, 22, 32, 40, 43]. It also highlights the superior cartilage repair quality achieved with the collagen‐augmented chondrogenesis technique compared with microfracture, regardless of age.

## LIMITATIONS

This study has several limitations. First, 90% of patients in the three groups were female. This high proportion of female patients is a common characteristic of Asian knee OA patients [15, 26, 29, 30, 41, 42]. Second, each of the three groups had a small sample size. Therefore, further studies with larger sample sizes are warranted. Third, the follow‐up period was short (2 years). Although the results of our study are meaningful, as previous studies have confirmed the effect of age on surgical outcomes even within 2 years [16, 18, 20], a longer follow‐up period is needed to clarify the effect of age on the outcomes of collagen‐augmented chondrogenesis. Fourth, this was a retrospective study with limitations, including potential selection bias and lack of randomisation. There were few patients in the microfracture group (Group 3) with 1‐year MRI results relative to the number of patients because this was a retrospective study; however, as many MRIs as possible were used for statistical analysis between the three groups. Fifth, although ICRS grades 3 and 4 may have different treatment policies or treatment outcomes [3], this study applied the same microfracture or collagen‐augmented chondrogenesis technique to patients without an ICRS grade 3 or 4 distinction. In this study, when comparing the WOMAC scores between ICRS 3 and 4 in each of the three groups, there were no significant differences in WOMAC scores between ICRS 3 and 4 patients in each group before and after surgery. Finally, the study's biggest limitation was that patients with HTO were included in all three groups. It might be difficult to judge the effect of the microfracture itself because simultaneous administration of HTO could affect the clinical features and cartilage regeneration. Despite these limitations, this is the first study to investigate the association between cartilage repair procedures and patient age.

## CONCLUSION

The collagen‐augmented chondrogenesis technique groups showed an improved quality of cartilage repair compared to the microfracture group, regardless of patient age. Compared to simple microfracture treatment, there were no differences in clinical outcomes between the patient groups, related to age.

## AUTHOR CONTRIBUTIONS

Man Soo Kim and Yong In both contributed to the literature search, conceptualisation, statistics analysis, supervision, validation and manuscript preparation. Keun Young Choi, Ryu Kyoung Cho, Hyuk Jin Jang, Dong Ho Kwak, Sung Cheol Yang and Seung Taek Oh contributed to the data collection and analysis. Man Soo Kim contributed to manuscript preparation. Yong In contributed to supervision and final approval of the draft version.

## CONFLICT OF INTEREST STATEMENT

The authors declare no conflict of interest.

## ETHICS STATEMENT

The study was approved by the Board of Research Ethics of Catholic University of Korea, Seoul Saint Mary's Hospital Research Ethics Committee (IRB KC22RASIO584). Informed consent was obtained from all individual participants included in the study.

## Data Availability

Data supporting this article are available from the corresponding author upon reasonable request.
